# Gray matter volume alterations in de novo Parkinson's disease: A mediational role in the interplay between sleep quality and anxiety

**DOI:** 10.1111/cns.14867

**Published:** 2024-07-19

**Authors:** Guixiang He, Xiaofang Huang, Haihua Sun, Yi Xing, Siyu Gu, Jingru Ren, Weiguo Liu, Ming Lu

**Affiliations:** ^1^ Department of Neurology Affiliated Nanjing Brain Hospital, Nanjing Medical University Nanjing China; ^2^ The Yancheng School of Clinical Medicine of Nanjing Medical University Yancheng Third People's Hospital Yancheng China; ^3^ Jiangsu Key Laboratory of Neurodegeneration, Department of Pharmacology Nanjing Medical University Nanjing China

**Keywords:** anxiety, gray matter volume, Parkinson's disease, sleep quality

## Abstract

**Objective:**

Parkinson's disease (PD) is increasingly recognized for its non‐motor symptoms, among which emotional disturbances and sleep disorders frequently co‐occur. The commonality of neuroanatomical underpinnings for these symptoms is not fully understood. This study is intended to investigate the differences in gray matter volume (GMV) between PD patients with anxiety (A‐PD) and those without anxiety (NA‐PD). Additionally, it seeks to uncover the interplay between GMV variations and the manifestations of anxiety and sleep quality.

**Methods:**

A total of 37 A‐PD patients, 43 NA‐PD patients, and 36 healthy controls (HCs) were recruited, all of whom underwent voxel‐based morphometry (VBM) analysis. Group differences in GMV were assessed using analysis of covariance (ANCOVA). Partial correlation between GMV, anxiety symptom, and sleep quality were analyzed. Mediation analysis explored the mediating role of the volume of GMV‐distinct brain regions on the relationship between sleep quality and anxiety within the PD patient cohort.

**Results:**

A‐PD patients showed significantly lower GMV in the fusiform gyrus (FG) and right inferior temporal gyrus (ITG) compared to HCs and NA‐PD patients. GMV in these regions correlated negatively with Hamilton Anxiety Rating Scale (HAMA) scores (right ITG: *r* = −0.690, *p* < 0.001; left FG: *r* = −0.509, *p* < 0.001; right FG: *r* = −0.576, *p* < 0.001) and positively with sleep quality in PD patients (right ITG: *r* = 0.592, *p* < 0.001; left FG: *r* = 0.356, *p* = 0.001; right FG: *r* = 0.470, *p* < 0.001). Mediation analysis revealed that GMV in the FG and right ITG mediated the relationship between sleep quality and anxiety symptoms, with substantial effect sizes accounted for by the right ITG (25.74%) and FG (left: 11.90%, right: 15.59%).

**Conclusion:**

This study has shed further light on the relationship between sleep disturbances and anxiety symptoms in PD patients. Given the pivotal roles of the FG and the ITG in facial recognition and the recognition of emotion‐related facial expressions, our findings indicate that compromised sleep quality, under the pathological conditions of PD, may exacerbate the reduction in GMV within these regions, impairing the recognition of emotional facial expressions and thereby intensifying anxiety symptoms.

## Graphical abstract

Compared to HCs and NA‐PD patients, A‐PD patients exhibited significantly reduced GMV in the fusiform gyrus and right inferior temporal gyrus. Our findings indicate that compromised sleep quality, under the pathological conditions of PD, may exacerbate the reduction in GMV within these regions, impairing the recognition of emotional facial expressions and thereby intensifying anxiety symptoms.

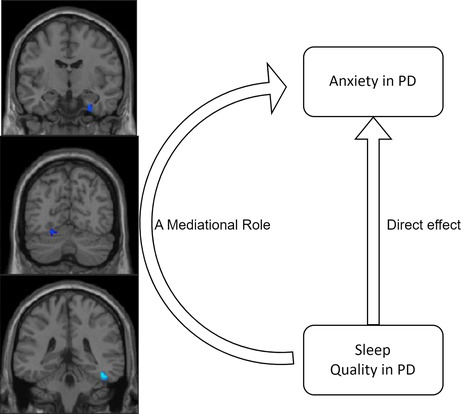

## 1 INTRODUCTION

Parkinson's disease (PD) is a progressive neurodegenerative disorder that is traditionally characterized by its motor symptoms. However, the disease's impact extends well beyond movement impairments, with a growing recognition of the significant burden imposed by non‐motor symptoms.^1^ Among these, anxiety, depression, cognitive impairment, and sleep disturbances are notably prevalent, affecting a considerable number of PD patients and significantly diminishing their quality of life.^2^ Anxiety in PD (A‐PD) can manifest in diverse forms, such as generalized anxiety disorder, panic disorder, and social anxiety, which not only heighten the complexity of the disease but also lead to increased healthcare utilization and caregiver strain.^3^ Furthermore, sleep disturbances in PD, which include insomnia, rapid eye movement sleep behavior disorder, and excessive daytime sleepiness, are equally debilitating. These sleep issues can further exacerbate cognitive decline and emotional distress, underscoring the importance of addressing non‐motor symptoms in the comprehensive management of PD.^4^

Neuroimaging studies have provided insights into the neural substrates of non‐motor symptoms in PD. Structural magnetic resonance imaging (MRI), especially voxel‐based morphometry (VBM), has consistently shown reductions in gray matter volume (GMV) in regions pivotal for emotional processing and sleep regulation, such as the amygdala, hippocampus, and prefrontal cortex, in PD patients with emotional disorders and in those with sleep disorders.^5^, ^6^, ^7^, ^8^ These findings suggest that GMV alterations may serve as a common neuroanatomical substrate for anxiety and sleep disturbances in PD. Functional MRI (fMRI) studies have further delineated aberrant functional connectivity (FC) within these regions, indicating potential disruptions in the neural networks associated with non‐motor symptom manifestation.^9^, ^10^, ^11^, ^12^ Despite significant progress, the majority of research has focused on patients in advanced stages of PD, where the effects of chronic dopaminergic medication and disease progression may confound neuroimaging findings.^13^ There is an urgent need to examine de novo PD patients to more accurately identify early neurobiological changes related to anxiety and sleep disturbances. Moreover, the relationship between anxiety and sleep disturbances in PD patients, despite their frequent co‐occurrence, remains underexplored, highlighting a notable gap in our understanding of the potential shared neural mechanisms.^14^, ^15^, ^16^

In this context, our study introduces a novel approach by examining the relationship between GMV and the interplay of anxiety and sleep quality in de novo PD patients. We hypothesize that A‐PD patients will exhibit GMV reductions in emotion‐related brain regions and that these changes will correlate negatively with anxiety severity, as measured by the Hamilton Anxiety Scale (HAMA), and positively with sleep quality, as assessed by the Parkinson's Disease Sleep Scale (PDSS).

Our study design strategically selected sleep quality as the independent variable and anxiety levels as the dependent variable, based on a targeted research objective and a thorough theoretical and empirical foundation. This decision was driven by our aim to investigate the nexus between anxiety symptoms and GMV in PD patients, emphasizing sleep quality's influential role. Literature reviews substantiated a strong link between sleep and anxiety, prompting our hypothesis that enhanced sleep could mitigate anxiety in PD.^17^, ^18^, ^19^ Utilizing a cross‐sectional design, we established a correlation between improved sleep and reduced anxiety levels in PD patients. Our mediation analysis dissected the pathways through which sleep quality could influence anxiety, reinforcing the selection of sleep as the independent variable. This decision is supported by clinical insights and theoretical evidence that recognize sleep quality as a modifiable factor antecedent to anxiety in PD.

The significance of this study lies in its potential to shed light on the neurobiological underpinnings of non‐motor symptoms in the early stages of PD. By elucidating the neural correlates of anxiety and sleep disturbances, this research may inform the development of targeted interventions that address these debilitating symptoms. Moreover, the study's findings may contribute to the refinement of neuroimaging biomarkers for A‐PD and sleep disturbances, facilitating early diagnosis and personalized treatment strategies.

## 2 MATERIALS AND METHODS

### 2.1 Participants

This study was conducted with the approval of the Medical Ethics Committee at the Affiliated Brain Hospital of Nanjing Medical University. All participants provided written informed consent. We recruited individuals diagnosed with de novo PD who visited our hospital's Neurology Department for movement disorder assessments between October 2018 and October 2022, as well as healthy controls (HCs) undergoing routine physical examinations.

Inclusion criteria for PD participants were: (1) meeting the 2015 Movement Disorder Society Clinical Diagnostic Criteria for Parkinson's disease (MDS‐PD Criteria)^20^; (2) an age range of 45–75 years; (3) capability to undergo MRI scanning and complete assessment scales; (4) right‐handedness; and (5) no history of anti‐PD medications, antidepressants, or anxiolytics. Exclusion criteria for PD participants included: (1) presence of significant structural brain lesions (e.g., cerebrovascular disease, severe head trauma, epilepsy, neurosurgical procedures) or other neurological or psychiatric conditions (e.g., schizophrenia, bipolar disorder); (2) secondary parkinsonism attributable to drugs or infections, or a clinical diagnosis of a parkinsonism‐plus syndrome; (3) Mini‐Mental State Examination (MMSE) scores ≤26; and (4) excessive head movement during MRI scanning (>2 mm), which could affect data analysis.

HCs were included if they: (1) matched PD participants in age, gender, and years of education; (2) were capable of undergoing MRI and scale assessments; and (3) were right‐handed. HC participants were excluded from the study if they: (1) had significant structural brain lesions or other neurological or psychiatric conditions; (2) were taking antidepressants or anxiolytics; and (3) had MMSE scores ≤26.

### 2.2 Clinical evaluation

All participants underwent comprehensive assessments, including brain MRI, clinical data collection, and neurological and psychiatric assessments. Demographic details such as age, gender, and years of education were recorded for each participant. Cognitive function was assessed using the MMSE, while the HAMA and the 24‐item Hamilton Depression Rating Scale (HAMD) were utilized to evaluate anxiety levels and depressive symptoms, respectively. For PD patients, additional data were collected regarding disease duration, motor status as measured by the Unified Parkinson's Disease Rating Scale part III (UPDRS III), and the modified Hoehn‐Yahr stage (H‐Y). Sleep quality was evaluated using the first three items of the PDSS.^21^, ^22^, ^23^ Based on prior research establishing a HAMA score of 11/12 as a sensitive and specific threshold for anxiety in PD,^24^ patients were categorized into A‐PD with HAMA scores ≥11 and NA‐PD with HAMA scores <11.

### 2.3 MRI data acquisition

All participants were scanned using a 3.0T Siemens Verio system with an 8‐channel phased‐array head coil at the Affiliated Brain Hospital of Nanjing Medical University. Participants were positioned supine, and foam padding was utilized to minimize the head movement and reduce motion artifacts. They were instructed to maintain stillness throughout the scanning session. Structural MRI data were acquired using a T1‐weighted three‐dimensional magnetization‐prepared rapid acquisition gradient echo (MPRAGE) sequence with the following parameters: repetition time/echo time (TR/TE) of 2530/3.34 ms, flip angle of 7°, field of view (FOV) of 256 × 256 mm, slice thickness/gap of 1.33 mm/0.5 mm, and a matrix size of 256 × 192, yielding 128 contiguous slices covering the entire brain. The duration of the MRI acquisition was approximately 8 min and 7 s.

### 2.4 MRI data preprocessing

The preprocessing of high‐resolution T1‐weighted MR images was conducted using VBM with the Computational Anatomy Toolbox (CAT12; http://dbm.neuro.unijena.de/cat12/), integrated within the Statistical Parametric Mapping (SPM12; http://www.fil.ion.ucl.ac.uk/spm/) software package. Initially, images were assessed for quality and realigned to the anterior commissure to correct for misalignment. Subsequently, the T1‐weighted images underwent a comprehensive processing workflow that included the application of the New Segment and DARTEL algorithms for image alignment and segmentation.^25^, ^26^ This resulted in the segmentation of the images into gray matter (GM), white matter (WM), and cerebrospinal fluid (CSF).^25^ The segmented images were then normalized to the Montreal Neurological Institute (MNI) space and resampled to a resolution of 2 × 2 × 2 mm^3^. Following rigorous quality control checks, the GM images were smoothed using an 8 mm full width at half maximum (FWHM) Gaussian kernel. The smoothed GM images were subsequently utilized to calculate the GM volume for between‐group analyses.

### 2.5 Statistical analyses

Demographic and clinical data were analyzed utilizing IBM SPSS Statistics, version 25.0 (SPSS Inc., Chicago, IL, USA). The normality of distribution for continuous variables was assessed using the Shapiro–Wilk test. Age across the three participant groups was compared using one‐way analysis of variance (ANOVA), while non‐parametric comparisons for years of education, MMSE scores, HAMD scores, and HAMA scores were conducted using the Kruskal–Wallis *H* test. Gender distribution among groups was evaluated with the Chi‐square test. Disease duration, UPDRS III scores, and modified H‐Y stage were analyzed using the Mann–Whitney *U*‐test. A two‐tailed *p*‐value of <0.05 was considered statistically significant. Normally distributed data were reported as mean ± standard deviation (SD), whereas non‐normally distributed data were presented as median (interquartile range, IQR).

To identify regions with significant GMV differences among the groups, analysis of covariance (ANCOVA) was employed, adjusting for gender, age, education, HAMD scores, and total intracranial volume (TIV) as covariates. Statistical significance was ascertained using a voxel‐wise threshold of *p* < 0.001 and a cluster‐wise threshold of *p* < 0.05, with corrections for multiple comparisons applied using Gaussian Random Field (GRF) theory. Regions exhibiting significant inter‐group GMV differences were identified using a mask, within which post hoc two‐sample *t*‐tests were conducted to further evaluate GMV differences, controlling for the aforementioned covariates and maintaining a significance level of *p* < 0.05 with Bonferroni correction.

We performed partial correlation analyses using SPSS 25.0 to investigate the relationship between the GMV of the FG and right ITG, HAMA scores, and sleep quality in PD patients. Gender, age, and education were adjusted as covariates to control for demographic influences. The statistical significance was set at *p* < 0.05, employing a two‐tailed test. Additionally, the study utilized a bootstrap resampling method to assess mediation effects, following established protocols in psychological research. Specifically, for our sample size under 500 individuals, we generated 5000 resamples using the percentile bootstrap approach, as recommended in prior literature.^27^ This method refines the estimation of confidence intervals for indirect effects, thereby strengthening the mediation analysis framework. In our model, sleep quality was designated as the independent variable, with anxiety levels as the dependent variable. Given the significant GMV differences between A‐PD and NA‐PD (PD patients without anxiety), the GMV was hypothesized as a potential mediator, offering insights into the interplay between brain morphology and psychological symptoms in PD.

## 3 RESULTS

### 3.1 Demographic and clinical data comparison

The demographic and clinical characteristics of the study cohort are presented in Table 1. A total of 116 participants were included, comprising 37 in the A‐PD group, 43 in the NA‐PD group, and 36 HCs. Intergroup comparisons revealed no significant differences in gender, age, educational level, or MMSE scores. Additionally, no significant disparities were observed between the A‐PD and NA‐PD groups concerning disease duration, UPDRS‐III scores, or modified H‐Y stage. However, the A‐PD group demonstrated significantly elevated HAMA and HAMD scores compared to the NA‐PD group (*p* < 0.001), and also exhibited reduced PDSS scores and poorer sleep quality (*p* < 0.001).

**TABLE 1:** Demographic and clinical characteristics of all participants.

Variables	A‐PD (*n* = 37)	NA‐PD (*n* = 43)	HC (*n* = 36)	*F*/*χ* ^2^/*H*/*Z*	*p*
Age (years)	58.65 ± 7.67	58.00 ± 7.44	58.94 ± 6.03	0.185	0.832^a^
Male sex, *n* (%)	16 (43.2)	23 (53.5)	18 (50.0)	0.851	0.654^b^
Education (years)	11.0 (8.5, 12.5)	12.0 (9.0, 12.0)	10.0 (8.0, 12.0)	0.369	0.831^c^
MMSE scores	28.0 (28.0, 29.0)	29.0 (28.0, 29.0)	29.0 (28.0, 30.0)	4.971	0.083^c^
HAMA scores	13.0 (12.0, 16.0)	1.0 (0, 2.0)	0 (0, 2.0)	78.194	<0.001^c^
HAMD‐24 scores	16.0 (12.5, 22.0)	3.0 (2.0, 5.0)	0 (0, 1.0)	82.458	<0.001^c^
Disease duration (years)	2.0 (1.0, 2.5)	1.0 (0.5, 2.0)	NA	−1.671	0.095^d^
PDSS scores	116.0 (92.0, 126.0)	142.0 (135.0, 146.0)	NA	−6.315	<0.001^d^
Sleep quality scores	19.0 (11.5, 23.0)	29.0 (25.0, 30.0)	NA	−6.646	<0.001^d^
UPDRS‐III scores	20.0 (15.0, 22.0)	16.0 (12.0, 21.0)	NA	−1.480	0.139^d^
Modified H‐Y stage	1.5 (1.0, 2.0)	1.5 (1.0, 2.0)	NA	−1.517	0.129^d^

*Note*: Data are presented as mean ± standard deviation, median (lower quartile and upper quartile) for continuous variables, or frequencies for categorical ones. For comparisons of demographics, ^a^ *p* values were obtained by one‐way analysis of variance (ANOVA) tests. ^b^ *p* value for the gender difference was obtained by chi‐square test. ^c^ *p* values were obtained by Kruskal–Wallis *H* test. ^d^ *p* value was obtained using the Mann–Whitney *U* test.

Abbreviations: A‐PD, PD patients with anxiety; HAMA, Hamilton Rating Scale for Anxiety; HAMD‐24, 24‐item Hamilton Rating Scale for Depression; HC, healthy control; H‐Y, Hoehn & Yahr staging scale; MMSE, Mini‐Mental State; NA, not applicable; NA‐PD, PD patients without anxiety; PDSS, Parkinson's Disease Sleep Scale; UPDRS‐III, Unified Parkinson's Disease Rating Scale–motor part III.

### 3.2 GMV comparison

**FIGURE 1:**
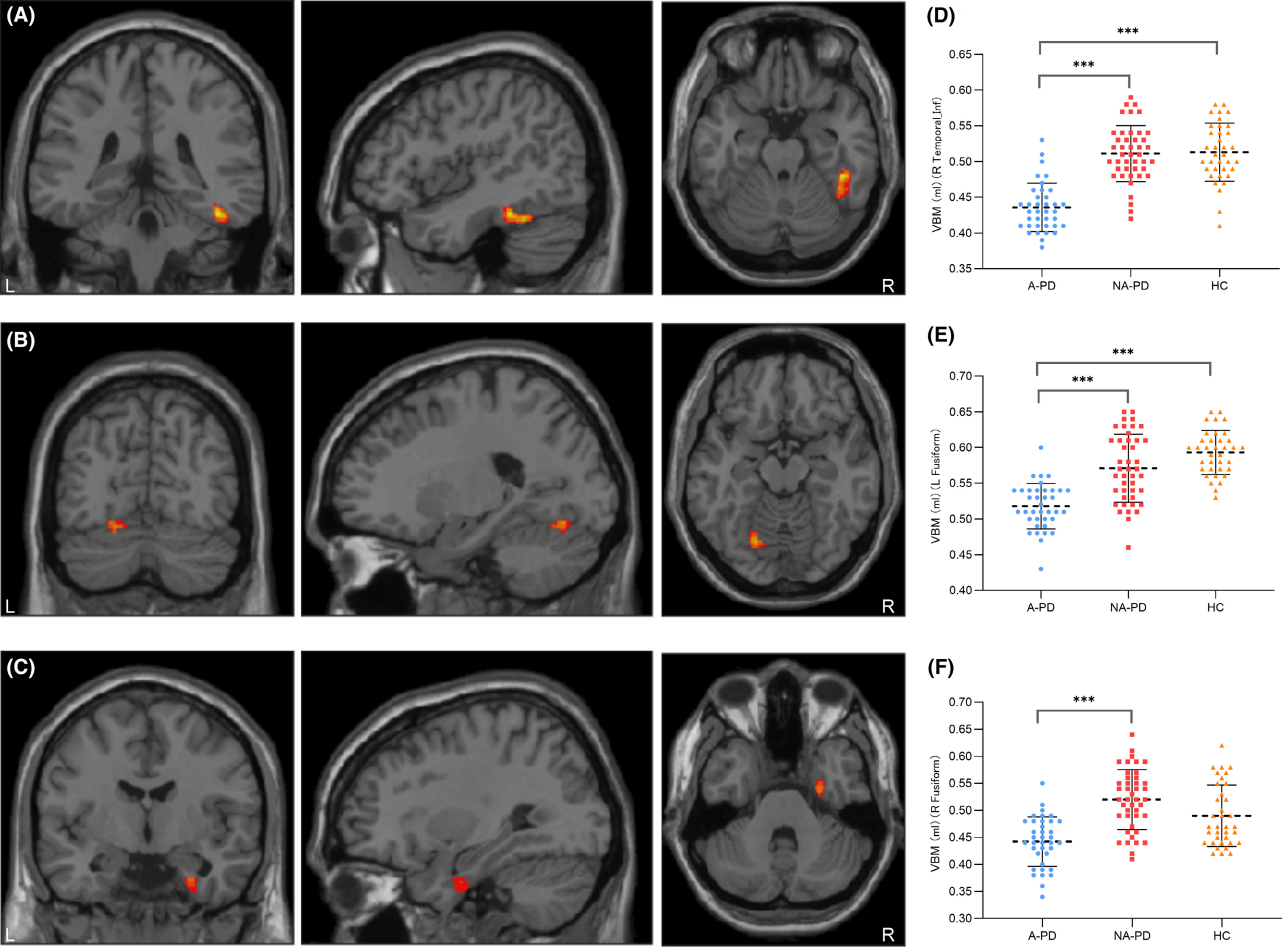
(A–C) GMV reduction regions in PD with anxiety (A‐PD) (*p* < 0.05, bofferoni correction). (A) Figure of the cluster with the peak point located in the right ITG. (B) Figure of the cluster with the peak point located in the left FG. (C) Figure of the cluster with the peak point located in the right FG. (D) Plot of the GMV distribution in the cluster with the peak point located in the right ITG. Post hoc analysis revealed a significantly decreased GMV in the A‐PD group compared with the PD patients without anxiety (NA‐PD) group (***p < 0.001) and the healthy control (HC) group (***p < 0.001). (E) Plot of the GMV distribution in the cluster with the peak point located in the left FG. Post hoc analysis revealed a significantly decreased GMV in the A‐PD group compared with the NA‐PD group (***p < 0.001) and the HC group (***p < 0.001). (F) Plot of the GMV distribution in the cluster with the peak point located in the right FG. Post hoc analysis revealed a significantly decreased GMV in the A‐PD group compared with the NA‐PD group (***p < 0.001), while there was no statistically significant difference compared with the HC group.

The A‐PD group exhibited reduced GMV relative to both the HC and NA‐PD groups, with significant decreases observed in the fusiform gyrus (FG) and the right inferior temporal gyrus (ITG). No significant GMV differences were detected between the NA‐PD and HC groups. For a detailed graphical representation of GMV reductions in A‐PD participants, refer to Table 2 and Figure 1.

**TABLE 2:** Comparison of GMV in the A‐PD group, NA‐PD group, and HC group.

Brain region (ALL)	Cluster size	*F*/*t*	Peak MNI coordinate		
			*X*	*Y*	*Z*
ANCOVA					
Temporal_Inf_R	137	14.125	45	−37.5	−22.5
Fusiform_L	40	10.679	−21	−70.5	−15
Fusiform_R	51	9.076	27	−9	−33
A‐PD VS NA‐PD					
Temporal_Inf_R	136	−9.125	45	−37.5	−22.5
Fusiform_L	34	−5.925	−21	−70.5	−15
Fusiform_R	51	−6.753	27	−9	−33
A‐PD VS HC					
Temporal_Inf_R	58	−8.840	43.5	−48	−24
Fusiform_L	40	−10.233	−22.5	−70.5	−13.5

*Note*: Three groups were compared using analysis of covariance, and two groups were compared using a two‐sample *t*‐test. Differences were statistically significant using GRF correction, voxel level *p* < 0.001, and cluster level *p* < 0.05.

Abbreviations: ANCOVA, analysis of covariance; A‐PD, Parkinson's disease with anxiety; GRF, Gaussian random field; HC, healthy control; L, left; MNI, Montreal Neuroscience Institute; NA‐PD, Parkinson's disease without anxiety; R, right.

### 3.3 Partial correlational analysis

**FIGURE 2:**
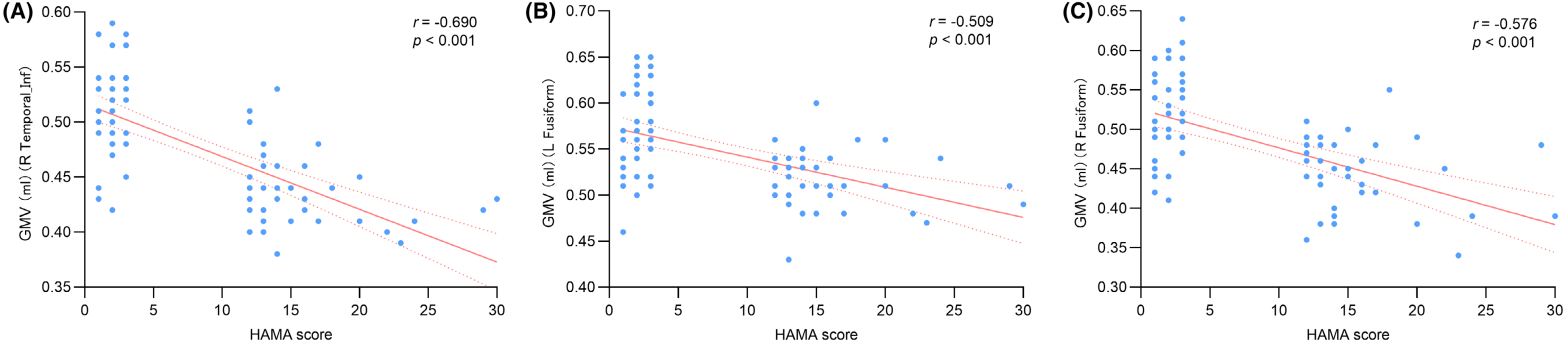
(A) The correlation between the anxiety problem score and the GMV of right ITG. (B) The correlation between the anxiety problem score and the GMV of left FG. (C) The correlation between the anxiety problem score and the GMV of right FG.

To investigate the behavioral relevance of the observed GMV changes in the FG and right ITG, partial correlation analyses were conducted between these GMV measures and HAMA scores. Partial correlation analyses within the PD group identified significant inverse correlations between HAMA scores and GMV in the right ITG (*r* = −0.690, *p* < 0.001), left FG (*r* = −0.509, *p* < 0.001), and right FG (*r* = −0.576, *p* < 0.001), as illustrated in Figure 2.

### 3.4 Mediation analysis

A robust inverse correlation between anxiety and sleep quality scores (*r* = −0.729, *p* < 0.001) was observed in our PD cohort, indicating that increased anxiety is linked to reduced sleep quality. Sleep quality scores correlated positively with GMV in key brain regions, including the right ITG (*r* = 0.592, *p* < 0.001), left FG (*r* = 0.356, *p* = 0.001), and right FG (*r* = 0.470, *p* < 0.001). These correlations were maintained after adjusting for the individual contributions of each brain region to anxiety and sleep quality. Specifically, the GMV of the right ITG was significantly associated with both sleep quality (*B* = 0.382, 95% CI [0.251, 0.512]; *p* < 0.0001) and HAMA score (*B* = −0.528, 95% CI [−0.737, −0.320]; *p* < 0.0001). The GMV of the left FG was also linked to sleep quality (*B* = 0.232, 95% CI [0.097, 0.367]; *p* = 0.001) and HAMA score (*B* = −0.402, 95% CI [−0.615, −0.188]; *p* < 0.0001). Additionally, the right FG GMV showed a significant relationship with sleep quality (*B* = 0.362, 95% CI [0.190, 0.533]; *p* < 0.0001) and HAMA score (*B* = −0.338, 95% CI [−0.504, −0.172]; *p* < 0.0001).

**FIGURE 3:**
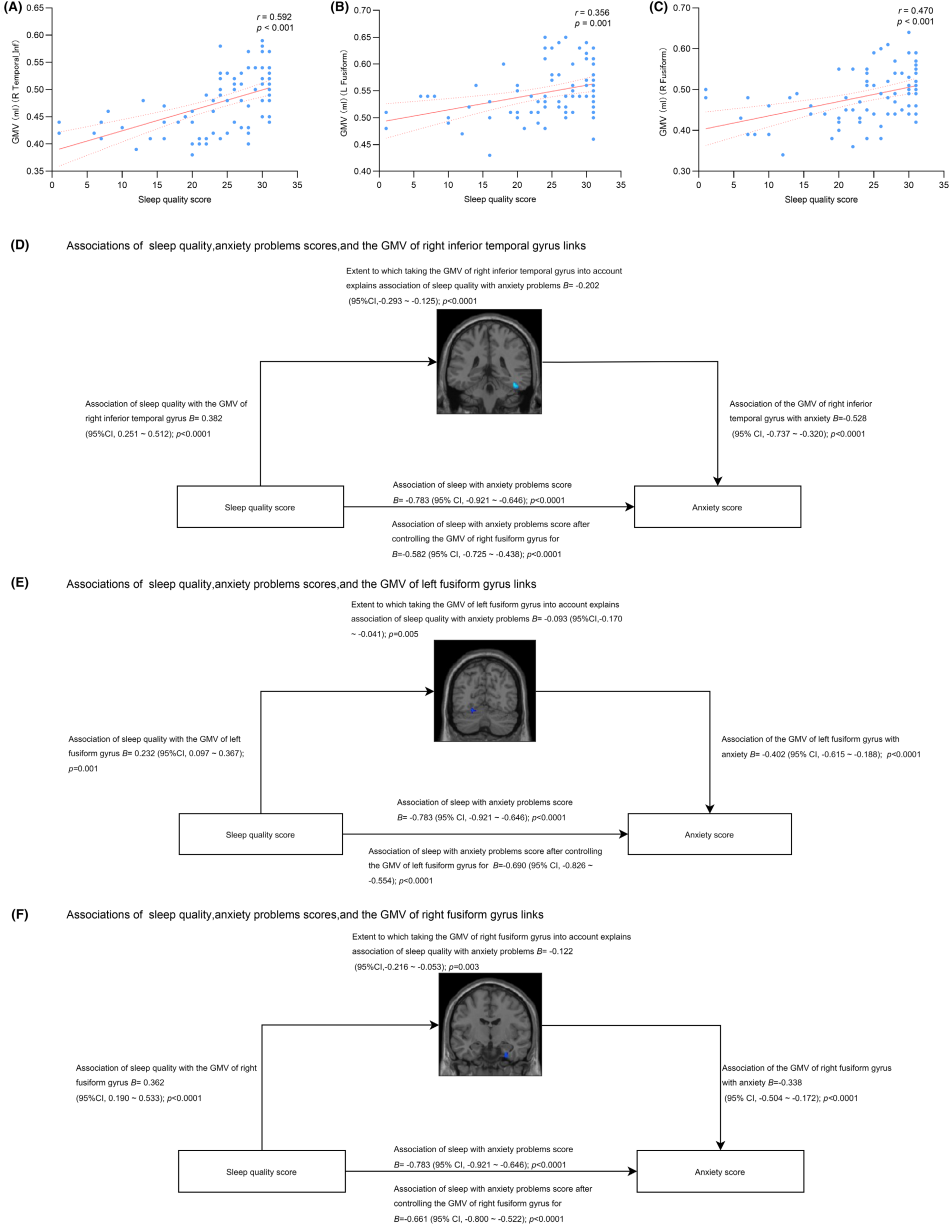
(A) The correlation between sleep quality scores and the GMV of right ITG. (B) The correlation between the sleep quality scores and the GMV of left FG. (C) The correlation between the sleep quality scores and the GMV of right FG. (D) The mediation implemented by the GMV of right ITG from poor sleep quality score on the anxiety problems (*B* = −0.202; *p* < 0.0001). (E) The mediation implemented by the GMV of left FG from poor sleep quality score on the anxiety problems (*B* = −0.093; *p* = 0.005). (F) The mediation implemented by the GMV of right FG from poor sleep quality score on the anxiety problems (*B* = −0.122; *p* = 0.003).

Mediation analysis revealed the mediating role of GMV in the relationship between sleep quality and anxiety. The right ITG GMV significantly mediated this relationship, accounting for 25.74% of the total effect size (*B* = −0.202, 95% CI [−0.293, −0.125]; *p* < 0.0001). The left FG GMV mediated 11.90% of the effect size (*B* = −0.093, 95% CI [−0.170, −0.041]; *p* = 0.005), and the right FG GMV mediated 15.59% (*B* = −0.122, 95% CI [−0.216, −0.053]; *p* = 0.003), as visualized in Figure 3.

## 4 DISCUSSION

The present study utilized VBM to investigate the GMV in patients with de novo A‐PD, revealing significant reductions in the FG and right ITG. These findings, which are negatively correlated with HAMA scores, suggest a neuroanatomical basis for A‐PD and highlight the potential mediating role of GMV in the relationship between sleep quality and anxiety. The results contribute to the growing body of literature that implicates structural brain changes in the manifestation of non‐motor symptoms in PD.

### 4.1 Advantages and limitations of VBM in PD assessment

VBM is recognized for its unbiased, whole‐brain analytical capabilities, which are particularly advantageous for the characterization of neurodegenerative diseases.^28^, ^29^ The technique's sensitivity to subtle and diffuse structural alterations is pivotal in delineating the neurodegenerative trajectory of such conditions.^30^ Although our study was constrained by a modest sample size, the observed GMV changes in the FG and ITG are noteworthy and consistent with the anticipated neuroanatomical patterns in A‐PD. It is important to acknowledge the intrinsic limitations of VBM, which include a lack of discernment between cortical thinning and alterations in surface area, as well as an absence of assessment regarding WM integrity.^31^ Nonetheless, when integrated with diffusion tensor imaging (DTI) and fMRI, which respectively offer insights into WM microstructural properties and brain activity, VBM can significantly augment the neurobiological characterization of A‐PD.^32^ This multimodal MRI strategy has the potential to clarify the complex interplay among brain structure, function, and connectivity, particularly in the context of anxiety associated with PD.^32^, ^33^

### 4.2 FG and right ITG GMV reductions in A‐PD

The FG and ITG are regions known to be integral in facial emotion recognition and emotional processing.^33^, ^34^, ^35^ Our study's detailed analysis has identified GMV changes within these regions that are specific to A‐PD patients and significantly correlated with anxiety severity. Consistent with existing literature, alterations in GMV and reductions in cortical thickness (CT) within the FG have been observed in patients with anxiety disorders without PD.^36^, ^37^, ^38^ Furthermore, an increase in the fractional amplitude of low‐frequency fluctuations (fALFF) within the FG, coupled with elevated neural activity, has been reported, which further supports the notion that these regions play a significant role in the pathophysiology of anxiety.^39^ Functional connectivity assessments have shown augmented connections between the FG and the amygdala in individuals experiencing anxiety, suggesting a disruption in the neural networks that regulate emotional responses.^40^, ^41^, ^42^ The ITG, with its extensive connections to limbic regions through the inferior longitudinal fasciculus (ILF), plays a significant role in emotional regulation.^43^ Heightened functional connectivity between the ITG and other brain regions such as the orbitofrontal cortex (OFC), amygdala, hippocampus, and parahippocampal gyrus has been associated with increased anxiety in PD patients.^12^ Our findings of reduced GMV in the FG and ITG, which mediate the relationship between poor sleep quality and heightened anxiety symptoms, provide a neurobiological foundation for understanding the interplay between these non‐motor symptoms.

### 4.3 Implications for PD pathology

PD is traditionally defined by the presence of Lewy bodies and the degeneration of dopaminergic neurons. Braak's hypothesis posits that during stages 3 and 4 of PD, Lewy body pathology extends beyond the substantia nigra to encompass the limbic system and temporal cortex,^44^ resulting in structural and functional impairments in these regions. However, our study emphasizes the neurobiological basis of non‐motor symptoms such as anxiety, which significantly contribute to the disease burden. The inverse relationship between GMV in the FG and ITG and anxiety levels suggests that individuals with more severe anxiety may exhibit structural alterations in these areas, potentially due to the pathological changes associated with PD. The FG's role in processing emotional facial information and the ITG's contribution to higher cognitive functions and emotional regulation make these regions critical for understanding the manifestation of anxiety in A‐PD.^45^, ^46^

The structural and functional alterations observed in the FG and ITG may arise from neuronal dysfunction and changes in the glial milieu due to Lewy body accumulation.^47^, ^48^, ^49^, ^50^ Such alterations could impair the visual processing of emotional cues and the management of social information, potentially triggering anxiety in response to social situations. Our findings contribute to the development of treatment methods targeting non‐motor symptoms in order to improve the overall quality of life for PD patients.

### 4.4 Partial mediation of sleep quality and anxiety by GMV reductions in FG and right ITG in PD

The neural interplay between sleep quality and anxiety in PD remains a vastly underexplored area. Anxiety, often comorbid with insomnia, affects approximately 50% of individuals with anxiety disorders, leading to sleep disruptions that can trigger or worsen anxiety symptoms.^18^ In PD, pathological changes significantly alter sleep architecture, characterized by disruptions in sleep quality, prolonged sleep onset latency (SOL), and diminished sleep efficiency (SE) and slow‐wave sleep (SWS).^51^ A meta‐analysis of six observational studies has reinforced the notion that insomnia is a robust predictor of anxiety episodes, with a pooled odds ratio of 3.23.^52^ Furthermore, sleep fragmentation has been demonstrated to intensify anxiety‐related symptoms.^53^

Our study addresses this void in the literature by establishing a significant negative correlation between sleep quality and anxiety levels in A‐PD patients, a correlation mediated by reductions in GMV within the FG and right ITG. These findings are congruent with an emerging consensus that structural brain changes are integral to the non‐motor manifestations of PD.^54^, ^55^ Our research supports the idea that primary insomnia and sleep deprivation are linked to alterations in critical brain regions that govern emotional regulation, including the amygdala, prefrontal cortex, hippocampus, FG, and ITG.^56^, ^57^, ^58^, ^59^, ^60^, ^61^ The FG and ITG, as our mediation analysis suggests, are pivotal in the nexus between sleep quality and anxiety in A‐PD patients. The ramifications of our findings are significant: they contribute to the neurobiological framework of A‐PD and imply that interventions focused on enhancing sleep quality may be a viable approach for mitigating anxiety symptoms. The identified GMV reductions in the FG and ITG, acting as mediators, present these areas as potential targets for non‐pharmacological interventions, such as transcranial magnetic stimulation (TMS).

A key strength of our study lies in the inclusion of de novo PD patients, enabling an examination of the disease's early manifestations unconfounded by long‐term medication effects. Our application of VBM for GMV analysis across the entire brain offers a thorough evaluation of structural changes. Despite this, the lack of significant GMV differences between NA‐PD and HC groups is likely due to the early disease stage, indicated by a low median UPDRS III score of 16 and minimal variance in anxiety and cognitive measures within our cohort. Furthermore, the absence of detected changes in the frontal cortex and amygdala may be attributed to several factors: the short illness duration in our patient cohort may not yet have resulted in other observable GMV changes; the stringent Bonferroni correction applied with GRF theory for multiple comparisons may have increased the risk of Type II errors, potentially obscuring subtle changes; and our analysis, focused solely on GMV, did not account for functional metrics, possibly overlooking neurological changes associated with anxiety that are not reflected in GMV. In conclusion, our findings highlight the significant role of the FG and right ITG in the sleep‐anxiety relationship in A‐PD patients. These results are instrumental for developing targeted interventions aimed at enhancing sleep quality and mitigating anxiety symptoms. Future studies should explore non‐pharmacological treatment potential focusing on these brain regions and examine the longitudinal impact of sleep quality on anxiety symptoms and brain structure in A‐PD patients.

### 4.5 Limitations

This study acknowledges several limitations. Firstly, the relatively small sample size may limit the generalizability and statistical power of the findings. Potential biases in the sample selection process could also affect the interpretation of the results. Secondly, while VBM provided a comprehensive assessment of the GM structure, it did not differentiate between changes in cortical folding and thickness, which could offer more nuanced information. Thirdly, the cross‐sectional design of our study limits our ability to infer causality between GMV changes and anxiety symptoms. Future research should employ larger sample sizes, longitudinal study designs, and more comprehensive assessment methods to validate and expand upon these findings.

## 5 CONCLUSION

In conclusion, our study indicates that A‐PD patients exhibit significant GMV reductions in the FG and right ITG, which mediate the relationship between sleep quality and anxiety. These neuroanatomical alterations provide morphological evidence for the interplay between sleep quality deficits and anxiety in PD patients. Interventions aimed at improving sleep, such as transcranial direct current stimulation (tDCS) and TMS, may hold therapeutic potential for alleviating anxiety symptoms. Future research should conduct longitudinal studies to track the relationship between GMV changes and anxiety symptoms and explore the evolution of these changes with disease progression and therapeutic interventions.

## CONFLICT OF INTEREST STATEMENT

The authors certify that the study was executed without any entanglements in commercial or fiscal arrangements, ensuring that there was no potential for conflicts of interest.

## Data Availability

The data that support the findings of this study are available on request from the corresponding author. The data are not publicly available due to privacy or ethical restrictions.
